# Data Collection Framework for Energy Efficient Privacy Preservation in Wireless Sensor Networks Having Many-to-Many Structures

**DOI:** 10.3390/s100908375

**Published:** 2010-09-08

**Authors:** Hayretdin Bahşi, Albert Levi

**Affiliations:** 1 Turkish National Research Institute of Electronics and Cryptology, Gebze, Kocaeli, Turkey; 2 Faculty of Engineering and Natural Sciences, Sabancı University, Orhanlı - Tuzla, 34956 Istanbul, Turkey; E-Mail: levi@sabanciuniv.edu

**Keywords:** privacy preserving data publishing, k-anonymity, wireless sensor networks

## Abstract

Wireless sensor networks (WSNs) generally have a many-to-one structure so that event information flows from sensors to a unique sink. In recent WSN applications, many-to-many structures evolved due to the need for conveying collected event information to multiple sinks. Privacy preserved data collection models in the literature do not solve the problems of WSN applications in which network has multiple un-trusted sinks with different level of privacy requirements. This study proposes a data collection framework bases on *k*-anonymity for preventing record disclosure of collected event information in WSNs. Proposed method takes the anonymity requirements of multiple sinks into consideration by providing different levels of privacy for each destination sink. Attributes, which may identify an event owner, are generalized or encrypted in order to meet the different anonymity requirements of sinks at the same anonymized output. If the same output is formed, it can be multicasted to all sinks. The other trivial solution is to produce different anonymized outputs for each sink and send them to related sinks. Multicasting is an energy efficient data sending alternative for some sensor nodes. Since minimization of energy consumption is an important design criteria for WSNs, multicasting the same event information to multiple sinks reduces the energy consumption of overall network.

## 1. Introduction

Recent technological advances produce low cost wireless sensors for observing many physical phenomena of the world like temperature, humidity *etc.* As wireless sensor technology takes progress, the missions of WSNs get complicated; they are used in human, enemy, habitat, structure or traffic monitoring applications. With the advent of wireless body networks, applications for health monitoring of patients outside the hospitals or home-caring of elderly people have been designed and implemented widely.

Recent WSNs have begun to collect much more information than simple WSNs observing temperature or humidity value of an environment. In an addition to spatio-temporal information of an event, especially in object monitoring applications, other attributes of monitored objects are gathered by sensors. For example, traffic monitoring applications collect velocity, direction and size information of a vehicle.

As the complexity of wireless sensor applications increase, structures of WSNs have evolved in order to meet the new application requirements. WSNs generally have many-to-one structure so that sensors collect event information from the area and send to a unique sink. Some recent sensor applications have begun to use many-to-many structure, which means that there exists multiple sinks in the deployed environment. WSN application may need to send the same event information to different sinks rather than a unique sink. For example, in a home-caring application for elderly people, information about the elderly person can be sent to a family member and a nurse at the same time.

As the capability of WSNs improves, privacy preserving is becoming one of the major problems in these networks. Huge amount of information about an individual is collected and distributed. Individuals generally need to restrict the details of personal information. Therefore, countermeasures for privacy threats have to cover both needs: enable data collection and restrict the storage of some private parts.

Network and database communities approach the privacy problem from different aspects. The network community mostly thinks that hiding sender and/or receiver in network communications is the prominent privacy problem. Privacy threat models presented by this community are based on concealing communication profiles against traffic analysis attacks. These attacks focus on external threats like global or local eavesdropping.

On the other hand, database community uses “data” per se as the subject of privacy. They bring solutions for privacy preserved storage and sharing of data. However, privacy models are not suitable for the actual needs of network applications, where data is gathered from users and relayed to data collectors. There are some models that fully trust data collection parties, which are not very realistic in most of the cases. There are some other models that consider data collector as an un-trusted entity. However, in such models, privacy preservation is provided by sending perturbed data to the data collector, which limits the types of analysis that can be performed by data collector.

Also, data collection models do not meet the requirements of having many data collectors with different privacy levels, which may be the case in a WSN. This issue has to be dealt by WSN designers.

In most of the WSNs, minimization of energy consumption is one of the primary criteria due to limited battery capacity or unavailability of battery replacements. All other security countermeasures as well as the privacy preserving solutions have to perform their works with minimum energy.

In this study, privacy preserving data collection framework is proposed for WSNs. The framework is based on a network model which has multiple un-trusted sinks. Privacy requirement level of each sink is assumed to be different from each other, which can be a realistic scenario in recent WSN applications. Our proposed framework meets all the privacy requirements while consuming low amount of energy.

This paper is organized as follows: In Section 2, motivation of the study and some background information are given. This section also includes the description of threat/network model and statement of our contributions. Section 3 discusses the details of proposed anonymization method. Section 4 shows the experimental results of simulations. Literature review of the topic is presented in Section 5 Section 6 concludes the study.

## 2. Motivation and Background

Privacy is the ability of an individual or group to decide which information about themselves should not be disclosed or which information would be revealed to whom. Therefore, a privacy framework have to use methods which can be easily adapted to different requirements of applications and users.

The wide usage of WSNs in monitoring applications makes people’s life easy but they may cause violation of privacy. Untrusted parties can access the collected information by eavesdropping, physically capturing the sensors, or bypassing authentication to remotely access sensors or central databases [1]. Data encryption, key distribution and authentication mechanisms can limit the effects caused by these types of threats. Applicability of these mechanisms are not straightforward in WSNs, due to limitations like physical capturing possibility of cryptographic credentials or limited battery usage. Many studies proposed various methods in order to solve these security problems under the limitations of WSNs [2,3].

Restricting the details of information gathered by WSN is required for limiting the privacy risks originated from untrusted parties. On the other side, privacy requirements of individuals also force the designers of WSNs to carry out some restrictions for data shared with trusted parties. These trusted parties are actually sinks in WSNs. There are mainly two reasons for these types of requirements: First, the trusted party can be captured physically by un-trusted parties. Especially in applications where attackers have high motivation or where major vulnerabilities exist, including lack of physical security, data shared with trusted parties have to obey some privacy criteria. Enemy tracking, health monitoring, or wild-life monitoring applications are examples for these types of applications. The second reason is that individuals generally want to hide detail information of their personal lives directly from the trusted party. For example, in applications where health monitoring is performed outside the hospital, individuals do not want their exact location and time information to be sent to hospital particularly in non-urgent times. However, they may want to weaken their privacy requirements in urgent times in order to get appropriate medical helps. If there are many sinks in WSN, requirements of individuals may change for each sink as well. Revisiting health monitoring applications, information about individuals may be sent to the family members as well as to hospital. In these applications, individuals may be willing to share more detail information with their family members but not necessarily with the hospital.

### 2.1. Privacy Preserving Data Publishing *vs*. Privacy Preserving Data Mining

Many studies about data privacy has been done in the database field under the name of “privacy preserving data publishing” [4]. The main aim is to provide privacy of data tables which are exchanged by other parties. At first glance, it may be assumed that privacy problem can be easily solved by stripping off the attributes which identifies individuals like name, social security number, *etc.* However, it is shown that it is possible to identify the owner of a record by using the residual data and other public information sources. This attack is called “re-identification attack” [5]. Re-identification attack is based on the assertion that some attributes, called quasi-identifiers, can easily help to identify the individuals although they do not uniquely identify them. Anonymity, which is defined as being not identifiable of an individual within set of individuals [6], is used as a privacy criterion in order to make data resistant to “re-identification attacks”. “*k*-anonymity” brings a specific restriction to anonymity so that an individual is hidden among at least *k* other individuals [5]. Quasi-identifier attributes are generalized or suppressed in order to meet the requirements of anonymity. In a generalization operation, attribute is replaced by a more general one like replacement of birth data “04.05.1977” by 1977. One attribute or all attributes of a record are deleted in a suppression operation. These operations cause information loss so anonymity solutions intrinsically try to solve a trade-off between information loss and privacy. They try to cause minimum information loss while achieving the required level of privacy.

Privacy preserving data mining is another major field in privacy studies [7]. Privacy preserving data mining techniques perturb the original data so that application of data mining techniques to perturbed data will give the same accurate mining results. However, the party that obtains perturbed data has no possibility to obtain the original data that may violate the privacy of data owners. Some attributes of data records may be modified with false data, they may be swapped among different records or totally artificially created new records can be inserted to original data sets at the data owner side.

Privacy preserving data publishing techniques do not change the truthfulness of data at record level. They try to collect information as much as possible. They aim to provide data for widespread categories of data analysis tasks. In situations where the details of data analysis that would be performed by data collector party is not known in advance or there is a huge need for performing various types of analysis tasks at data collector side, data publishing techniques are preferred. The proposed framework in this paper is based on privacy preserving data publishing.

### 2.2. Privacy Preserving Data Collection Models

Studies on privacy problem mostly concentrated on achieving collection and sharing of data under the required privacy constraints in order to make efficient knowledge-based decisions. Data collection refers to the conveying of user data from data owners to a central data collector. In some situations, data collector may share this data with other third parties. Privacy preserving data collection is modelled according to the trust level between data owner and data collector party. These models are categorized into two categories, *trusted data collection model* [4] and un-trusted data collection model [8].

**Figure 1.**
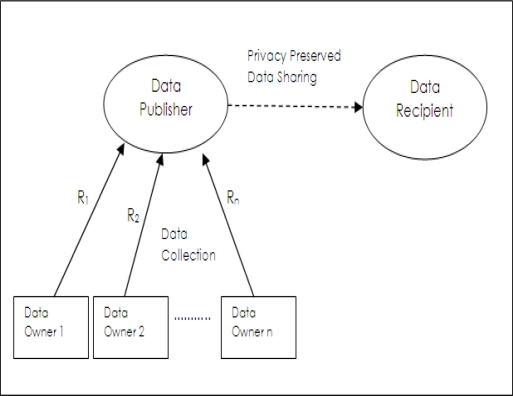
Trusted data collection model.

In trusted data collection model, [4] as shown in Figure 1, data is collected from data owners by data collector party named as *data publisher*. Data publisher shares data with *data recipient* who will actually use it for performing a required data analysis task. Here, data collection and data sharing are separate operations. A generic example is the application where hospitals share medical records with medical research institutions. In this example, data owners are patients, data publishers are hospitals and data recipients are medical research institutions. Data publishers collect all the details of records of data owners (*R*_1_ *R*_2_,···*R_n_*) but they are required to share privacy preserved data with data recipients. Data owners do not trust data recipient parties; however, they are required to fully trust data publishers in completing privacy preserving operations. Also data owners have to be sure that their data is not maliciously or unintentionally used for illegal duties by staff of data publishers.

An intrinsic assumption of this model is that data publisher does not know the details of analysis or data mining tasks which will be performed by data recipient. This may be due to situation that data publisher lacks technical expertise in the corresponding analysis methods or data publisher does not even know what type of analysis will be done on shared data in data recipient part. Privacy preserving data publishing methods are used to cover the requirements of this model.

**Figure 2.**
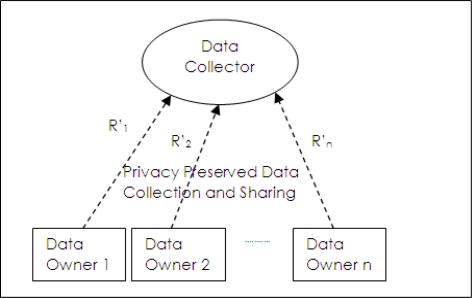
Untrusted data collection model.

In un-trusted data collection model as shown in Figure 2, data owners send their records to data collector but they do not trust it. Data collection and data sharing do not occur separately as in the trusted model. Privacy preservation has to be done at the data owner side and privacy preserved data (*R′*_1_, *R′*_2_,···*R′*_3_) are collected by data collectors. Privacy preserved data mining methods use this model.

In network applications that collect data from users, determination of analysis and mining tasks in the network design stage may limit the capability of this data collection system. Also, the requirements of analysis tasks may change with time. Therefore, in most of the time, privacy preserved data publishing methods are more useful in data collection applications. On the other side, these methods require a trusted data collector party or data publisher which collects centrally all the data. However, data owners may not fully trust them. Any person with malicious intent in the data collector site may have possibility to reach all the private data. Another possibility may be that due to inefficiency of security countermeasures at the data collector site or security problems during data collection operations, attackers can obtain private data. Data owners generally may want data to be privacy preserved before reaching these parties. Also, it is possible as in WSNs so that more than one sinks having different levels of privacy requirements may exist in the network. Therefore, a new model has to be devised so that privacy preserving data publishing methods can be applied for several un-trusted data collectors having different privacy needs. Since privacy preserving data publishing methods have to work on different users records for anonymization, they have to be collected somewhere before untrusted data collectors.

**Figure 3.**
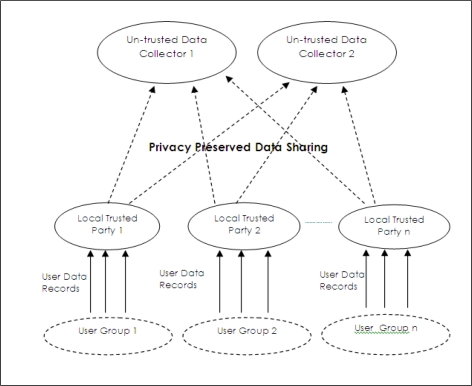
Distributed trusted data collection model.

A required model for privacy preserved data collection is shown in Figure 3. In this model, data owners send their data to local trusted parties. Appropriate privacy preserving operations are done at these local ones and privacy preserved data is sent to the data collector, which is untrusted for users. Since local data collectors are distributed, compromising one collector does not compromise all data in this model. If truthfulness of collected data records are allowed to change, privacy preserved data mining techniques can be applied as privacy preserving operations. On the other side, privacy preserved data publishing techniques are the most appropriate ones if data collectors do not tolerate changes in data truthfulness.

### 2.3. Threat and Network Model

Threat model is based on the requirement that data gathered by sinks has to have *k*-anonymity property in which different sinks require different amounts of *k* value. This means individuals who are owner of sensed data do not want sinks to identify their records among other records of *k* individuals within a data collected in a specified time-frame through the quasi-identifier fields of records. For simplicity, it is assumed that one event record is generated for each individual within that time-frame. The required privacy levels of each sink differs so that, suppose there are *n* sinks, each *i^th^* sink has a privacy level *p_i_* where each level requires to share *k_i_*-anonymous data with *i^th^* sink and inequality of *k*_1_ < *k*_2_... < *k_n_* is valid.

**Figure 4.**
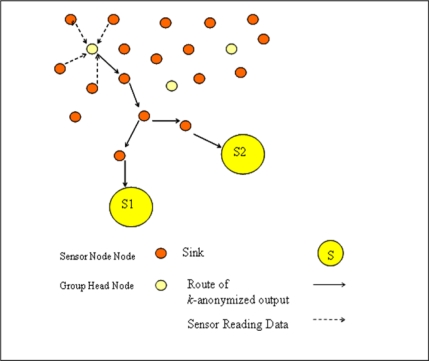
Network model.

Sensors are clustered in separate sensor groups according to sensor localizations where each group has a group head sensor. In our network model which is shown in Figure 4, each sensor conveys its readings to group heads, they *k*-anonymizes data and send this *k*-anonymized output to each sink.

If we compare network model of WSN with privacy preserved data collection model given in Figure 3, it can be stated that each group head sensor acts as a local trusted party and each sink is assumed an untrusted data collector.

Threat model does not directly address confidentiality of data in transit. However, data which is sent from group head sensors to sinks have *k_n_*-anonymity property. By eavesdropping on this data, attackers can obtain only *k_n_*-anonymous data.

Resistance to active attacks like malicious node attacks and false packet injection ones are out of the threat model scope.

### 2.4. Our Contribution

In this paper, *k*-anonymity notion is adapted as a privacy framework for WSN applications having multiple sinks. Collected event information is iteratively *k*-anonymized for all sinks each having different privacy levels. Encryption operation with appropriate key management schema is used in addition to generalization in order to meet the different requirements in one *k*-anonymized output. Achieving all privacy requirements in one output considerably decreases the energy consumption so that this output can be multicasted to multiple sinks instead of sending different outputs for each sink. Bottom-up clustering idea is used during *k*-anonymization process.

## 3. Proposed Anonymization Method, Iterative *k*-Anonymous Clustering Method (I*k*-ACM)

Proposed *k*-anonymization method, iterative *k*-Anonymization (I*k*-ACM), is based on the k-Anonymous Clustering Method given in [9]. I*k*-ACM basically produces a common *k*-anonymous data that meets each requirements of sinks by the help of encryption operation in addition to generalization operation. The main aim is to meet the privacy requirements with the minimum information loss. Sensor nodes directly transmit their sensed data to their group head sensors. After anonymization of data by using encryption and generalization at group head node, anonymized output is sent to each sink. Each sink decrypts the related encrypted parts and obtains data with required level of privacy.

I*k*-ACM is based on hierarchical bottom-up clustering idea. Quasi-identifier attributes of records are extracted from event data and they are fed as input vectors to iterative hierarchical clustering process. The basic idea is to partition the input vectors into clusters where each cluster has at least *k* vectors. During the clustering, each cluster generates a representative vector which contains common generalized values or encrypted versions of attributes of all vectors belonging to that cluster. Vectors of clusters are all replaced with this representative vector in the anonymous output. Since an appropriate distance function is used during clustering and appropriate generalizations, this replacement is expected to create minimum information loss. *k*-anonymization work takes place in group head sensors.

Subsection 3.1 explains how collected information is represented in our proposed method. In Subsection 3.2, distance metric which is used in the clustering process is described. Subsection 3.3 briefs how a common output is formed for meeting the needs of each sink. Subsection 3.4 gives the details of bottom-up clustering process which is the core of the proposed method. Subsection 3.5 investigates the computational complexity of method. Subsection 3.6 explains the details about the usage of proposed method in a WSN topology so that energy consumption is minimized.

### 3.1. Data Representation

Suppose input data is a table *T* having *m* attributes, *r* records. *T_ij_*, represents the *j*’th attribute of the *i*’th record where, {*i* : 1 ≤ *i* ≤ *r*} and {*j* : 1 ≤ *j* ≤ *m*}. Table *T* is represented by a set of bit strings *B*, where *B_ij_* is bit string representation of *j*’th attribute of *i*’th record. *k*’th bit of *B_ij_* is shown as *B_ij_*(*k*). Suppose that *j*’th attribute of table is categorical and there are *d_j_* distinct values. These values are indexed by *k* and shown as *V_j_*(*k*) where {*k* : 1 ≤ *k* ≤ *d_j_*}. Bit string of this categorical attribute has a size of *d_j_* and formed as follows:

$$\mathrm{I f}\;T_{\mathrm{ij}}=V_{j}(k)\;\;\mathrm{then}\;\;B_{\mathrm{ij}}(k)=1\;\;\mathrm{else}\;\;B_{\mathrm{ij}}(k)=0\;\;\mathrm{as}\;\;\forall k\;\;:\;\;0\leq k\leq d_{j},$$

If attribute is numerical, the range of attribute is divided into equal-sized intervals. Assume that *j*’th attribute is numeric and attribute range is divided into *d_j_* number of intervals. Each interval is indexed by *k*. Bit string representation of this numeric attribute has a size of *d_j_* and formed as follows:

$$\mathrm{I f}\;T_{\mathrm{ij}}\;\;\mathrm{intersects}\;\;\mathrm{with}\;\;k\prime \mathrm{th}\;\;\mathrm{interval},\;\;\mathrm{then}\;\;B_{\mathrm{ij}}(k)=1\;\;\mathrm{else}\;\;B_{\mathrm{ij}}(k)=0\;\;\mathrm{as}\;\;\forall k\;\;:\;\;0\leq k\leq d_{j}$$

Any attribute of original data before anonymization process has only one true bit in its bit string. However, the number of true bits increase due to the information loss during anonymization.

### 3.2. Information Loss Metric

Calculating the data loss of *k*-anonymous data is needed to predict the performance of our proposed method under different *k*-anonymity parameters. In our study, we use the entropy concept of information theory to measure the information loss [10]. The difference of entropies between the *k*-anonymous data and the original data constitutes the information loss. Suppose that *T* is the input data set having *r* records and *m* attributes, *B* is the bit string representation of this data set and *C* is the random variable that gets the probability value of an attribute value in a *k*-anonymous data entry being the actual attribute value in the original data. Assume that all the entries of *B* is normalized according to the number of bits having value “1” in that entry (from now on we refer “true bit” to a bit having value “1”) and normalized version forms data set *B̄*. A sample data set is shown in Table 1. Here, there are two records; each record has three attributes; each attribute is categorical and each has five distinct attribute values. Table 2 shows the normalized version of data. During normalization, each entry is divided by the number of true bits in the corresponding bit string entry.

Information loss of a data table *T*, *IL*(*T*), is equal to the conditional entropy, *H*(*C* | *B*). Here, conditional entropy gives the uncertainty about the prediction of the original attribute values of a record when we have the knowledge of corresponding *k*-anonymous bit strings of that record. Original data has only one true bit in each bit string because each original data entry corresponds to one attribute value. However, in *k*-anonymous data, each entry may have more than one attribute value and each attribute value is represented by an additional bit. Therefore, if an entry has only one true bit, that entry does not have information loss. In this situation, we have no doubt that this true bit is the true bit that comes from the original data. As the number of true bits increases, disorder of the data increases because it is harder to predict which one of them is the original true bit. Prediction gets harder because information is lost due to the increase in the number of true bits. Conditional entropy, which is used in order to calculate the disorder of the data, is a well measurement tool for the information loss. Conditional entropy *H*(*C* | *B*), which is equal to information loss of table *T*, *IL*(*T*), can be found as follows:

(1) $$\begin{array}{c} \mathrm{I L}(T)=H(C|B)=\Sigma_{B_{\mathrm{ij}}\in B}p\;(B_{\mathrm{ij}})H(C|B=B_{\mathrm{ij}})\\ \;\;\;\;\;\mathrm{I L}(T)=-\sum_{B_{\mathrm{ij}}\in B}p\;(B_{\mathrm{ij}})\sum_{k\in \{1..z\}}p(C=k|B_{\mathrm{ij}})\;\text{log}\;p(C=k|B_{\mathrm{ij}}) \end{array}$$

In Equation 1, it is assumed that each attribute is converted to bit strings having size *z*. This means all categorical attributes have *z* distinct attribute values and all numerical attributes have *z* number of interval ranges. Also, it is assumed that all *k*’s, where the equalities of *p*(*C* = *k* | *B_ij_*) = 0 are true, are excluded from the summation. *C* random variable can take values from the set {1..*z*}. Actually, *B̄* is calculated for finding the value of this random variable.

(2) $$p(C=k|B=B_{\mathrm{ij}})={\bar{B}}_{\mathrm{ij}}(k)\;\;\;\mathrm{for}\;\;\mathrm{each}\;\;k\;\;:\;\;1\leq k\leq z$$

In Equation 1, it is assumed that each record has equal probability to be chosen and each attribute of record has the same probability, therefore probability mass function of *j*’th attribute of *i*’th record, *p*(*B_ij_*), is calculated as $p(B_{\mathrm{ij}})=\frac{1}{m.r}$. Equation 1 can be rewritten as follows:

(3) $$\mathrm{I L}(T)=-\sum_{B_{\mathrm{ij}}\in B}\frac{1}{m.r}\sum_{k\in 1..z}{\bar{B}}_{\mathrm{ij}}(k).\text{log}\;{\bar{B}}_{\mathrm{ij}}(k)$$

Suppose that *F* is the array that contains the number of true bits of the bit string array *B*. Total number of true bits in *B_ij_* is *F_ij_*. Total number of elements in *B̄_ij_*(*k*) that has the value of $\frac{1}{F_{\mathrm{ij}}}$ is equal to *F_ij_*, and the rest is zero. Therefore, the second sum operation of Equation 3 yields the value, $\text{log}\frac{1}{F_{\mathrm{ij}}}$. The simplest equation for the information loss of data table *T*, *IL*(*T*), can be calculated as follows:

(4) $$\mathrm{I L}(T)=-\sum_{F_{\mathrm{ij}}\in F}\frac{1}{m.r}\text{log}\frac{1}{F_{\mathrm{ij}}}=\frac{1}{m.r}\sum_{F_{\mathrm{ij}}\in F}\text{log}F_{\mathrm{ij}}$$

### 3.3. Iterative Anonymization Model

In the WSN, there are *n* sinks, *m* sensors and *m.*(*n* − 1) symmetric encryption keys. Each sensor shares different key with each sink. Sharing is performed by physically deploying of keys to the sensors and to the sinks during network set-up phase of WSN. Since, it is not known which sensor will be group head during the operation of WSN, all sensors share different key with each sink.

*l_th_* sensor shares key with *p^th^* sink which is labelled as $e_{p}^{l}$. *i*^th^ sink contains list of the keys of all sensors as $e_{i}^{l},e_{i+1}^{l},\ldots ,e_{n-1}^{l}$ where 0 < l < *m*.

Each sensors store *n* − 1 keys. Practically, the number of multiple sinks may be between 2 and 5, sensors can easily store this number of keys in their limited memory. On the other side each sink contains the number of keys between 0 and *m.*(*n* − 1) keys. Since sinks can have memories with higher sizes, these number of keys can be stored in sinks easily.

In the literature, there exist various group head selection and group formation methods [11]. It is assumed that appropriate one used during formation of groups and selection of group head sensor. The keys are assumed to be physically distributed to the sinks and to the sensors before deployment.

**Figure 5.**
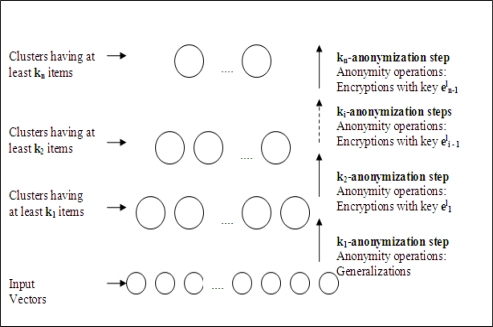
Steps of iterative anonymization.

In the proposed method, anonymization is completed in *n* iterative steps as shown Figure 5 in each group head sensor. Assume that we operate on *l^th^* sensor which is selected as a group head sensor during WSN operation. In the first step, by using only generalization operation, input data is *k*1-anonymized. In the second step, *k*1-anonymized data is *k*2-anonymized by encrypting the chosen data parts with $e_{1}^{l}$. For each *i*^th^ step to *n*^th^ step, anonymization is done by encryption using key, $e_{i-1}^{l}$. The output after *n*^th^ step is multicasted to all sinks.

After the arrival of anonymous data to sink from *l^th^* group head sensor, each sink decrypts the data with their keys. The resulting data after decryption actually has the level of privacy required for that sink. *i^th^* sink can only decrypt the data which is encrypted after the *i*^th^ iterations; because it has the corresponding keys. Data parts encrypted by the keys, $e_{1}^{l},e_{2}^{l},\ldots ,e_{i-2}^{l}$, cannot be decrypted, therefore they can be considered as suppression operations for that sink. 1st sink, which has to get data with lowest privacy criteria, can decrypt all the encrypted parts and the result data is actually *k*_1_-anonymized. On the other hand, *n*^th^ sink has no key and gathers data as *kn*-anonymized.

### 3.4. Bottom-Up Hierarchical Clustering Process

I*k*-ACM is based on forming clusters of input vectors iteratively. Input data to I*k*-ACM is represented as *T* and *i^th^* input vector is *T_i_*. Each cluster is numerated as $L_{j}^{h}$ in each iteration, *h*, where *j* is the index number of cluster. Input vector set of cluster $L_{j}^{h}$ is represented by $V_{j}^{h}$, the number of input vectors belonging to that cluster is $\left|V_{j}^{h}\right|$, and representative vector is $R_{j}^{h}$. Suppose that *k^th^* data item of representative vector is denoted as $R_{j}^{h}[k]$. Representative vector is actually the anonymized output of input vectors belonging to that cluster which is formed by generalization and encryption operations of some data parts of vectors. Assume that *D^h^* is distance matrix of iteration *h.* It contains distances between each cluster pairs.

Algorithm of I*k*-ACM is given in Algorithm 1. Hierarchical clustering process starts with the initialization phase given in second step of main function. In this phase each input vector constitutes separate cluster and that vector is also representative vector of the cluster. As given in the fourth phase of algorithm, iterations are divided into anonymization steps. Iterations continue until *n^th^*-anonymization step. In each iteration, by using the information loss metric described in Section 3.2, distances between each cluster are calculated. Distance between any two clusters is actually equal to the information loss that may occur if both clusters are merged. Two clusters having smallest distance, assume that clusters, $L_{s}^{h}$ and $L_{t}^{h}$, are chosen for merging. New bigger cluster, $L_{u}^{h+1}$ which contains the vector items of both clusters is formed and old two clusters are deleted. $\left|V_{u}^{h+1}\right|$ is equal to the sum of $\left|V_{s}^{h}\right|$ and $\left|V_{t}^{h}\right|$. For the first step of cluster operations (*k*1-anonymization stage), anonymity operation is generalization. In these generalization operations, $R_{u}^{h+1}[k]$, is equal to the XOR of $R_{s}^{h}[k]$ and $R_{t}^{h}[k]$. Encryption is used as an anonymity operation in all anonymization steps except *k*1-anonymization. Assume that two clusters, $L_{h}^{s}$, $L_{h}^{t}$ are chosen as the closest cluster pair at *h*^th^ iteration of I*k*-ACM and this iteration corresponds to (*i* + 1)*^th^*-anonymization step. The newly created cluster is labelled as $L_{h}^{\mathrm{st}}$ and *E_e_i−1__* (*x*) represents the encrypted output of input *x* with key *e_i_*_−1_. Formation of representative vector for newly created cluster, $R_{h}^{\mathrm{st}}$, is given in Algorithm 2.

**Figure 6.**
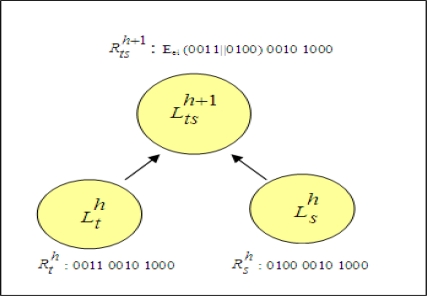
A sample case for cluster combination with encryption operations.

A sample cluster combination by encryption operation is shown in Figure 6. Assume that represented vectors of two closest clusters, $L_{t}^{h}$ and $L_{s}^{h}$ are ‘0011 0010 1000’ and ‘0100 0010 1000’, respectively. The values of vectors indicate that data records have three attributes each having four distinct attribute values. First bit strings of vectors, ‘0011’ and ‘0100’ are compared. Since they are not identical, concatenation of values are encrypted. This encryption result forms the first bit string of representative vector of newly created cluster, $L_{\mathrm{ts}}^{h+1}$. Second bit strings of clusters, ‘0010’ and ‘0100’, are identical. Therefore, second bit string of new cluster is also ‘0100’. By using the same method, third bit string is found as ‘1000’.

Clustering process occurs in each iteration of anonymization model described in Section 3.3. In that model, each iteration takes *k_i_*-anonymized output, clustering operations are completed until data is *k_i_*_+1_-anonymized. In the first iteration raw data is *k*_1_-anonymized by generalization operations. In the second one and the rest of all iterations data is anonymized to a higher level by encryption operations where different key is used in each iteration.

The anonymized output of I*k*-ACM is actually the representative vectors gathered at the end of *n^th^* steps. The formation of output is given in fifth and sixth phases of main function given in Algorithm 1.

**Algorithm 1.** I*k*-ACM Algorithm.

**Function** Cluster Combination
**Input :** parameter, *k*, distance matrix, *D^h^*, key, $e_{\mathrm{step}\_\mathrm{no}-1}^{l}$, anonymization step, step_no
**Output:** New cluster, $L_{u}^{h+1}$, updated distance matrix, *D^h^*^+1^
1. Find clusters, $L_{s}^{h}$, $L_{t}^{h}$, having minimum distance in distance matrix *D^h^*; create a new cluster $L_{u}^{h+1}$
2. $V_{u}^{h+1}\leftarrow V_{s}^{h}\cup V_{t}^{h}$
3. $\left|V_{u}^{h+1}|=|V_{s}^{h}|+|V_{t}^{h}\right|$
4. If *step_no* = = 1
For each *z^th^* bit string of representative vector, $R_{u}^{h+1}[z]=R_{s}^{h}[z]$**OR**$R_{t}^{h}[z]$
else
$R_{u}^{h+1}$ ← Function Form_Encrypted_Representative_Vector ( $L_{s}^{h}$, $L_{t}^{h}$, *e*_*step_no*−1_)
(Function is given in Algorithm 1.)
5. Remove clusters, $L_{s}^{h}$, $L_{t}^{h}$
6. Find the distance of $L_{u}^{h+1}$ to other clusters, update *D^h^*^+1^
**Main Function** I*k*-ACM
**Input :** Table, *T*, number of records, *r*, number of attributes, *m*, number of sinks, *n*, anonymization parameters *k*_1_, *k*_2_,..., *k_n_*, index of group head sensor, *l*
**Output:** Anonymized table, I*k*-ACM(*T*)
**Initialization**
1. *h* = 1
2. for all *i* where {*i* : 0 < *i* < *r*}
2.1. Create cluster array, $\left\{L_{i}^{1}\right\}$
2.2. Add record, *T_i_* to $V_{i}^{1}$
2.3. Set initial size of cluster, $\left|V_{i}^{1}\right|=1$
2.4. Initialize the representative vector, $R_{i}^{1}\leftarrow T_{i}$
2.5. Initialize the distance matrix *D*^1^ by using Equation 4
**Iterative steps for multilevel***k***-anonymization**
3. *step_no* = 1
4. while *step* _*no* ≠ *n*
4.1. while not for each cluster $\left|V_{i}^{h}\right|\geq k_{\mathrm{step}\_no}$
4.1.1 Call Function ClusterCombination (*k*, *D^h^*, $e_{\mathrm{step}\_\mathrm{no}-1}^{l}$,*step* _*no*)
4.1.2 h=h+1
4.2. step_no=step_no+1
**Form the output of I***k***-ACM**
5. I*k*-ACM (T) is initialized to empty set
6. for each cluster, $L_{s}^{h}$ in *L^h^* where {*s* : 0 < *s* < |*L^h^*|}
6.1. Append $R_{s}^{h}$ and $\left|V_{s}^{h}\right|$ to I*k*-ACM(*T*)

**Algorithm 2.** Forming of representative vector.

**Function** Form_Encrypted_Representative_Vector
**Input :** Representative vectors, representative vector, $R_{h}^{s}$, representative vector, $R_{h}^{t}$, key $e_{\mathrm{step}\_\mathrm{no}-1}^{l}$
**Output:**Representative vector of new cluster, $R_{h+1}^{u}$
For each attribute *m*
if $R_{h}^{s}[m]=R_{h}^{t}[m]$ then
$R_{h+1}^{u}[m]=R_{h}^{t}[m]$
else
$R_{h+1}^{u}[m]=E_{e_{\mathrm{step}\_no-1}^{l}}(R_{h}^{s}[m]||R_{h}^{t}[m])$

### 3.5. Complexity Analysis of I*k*-ACM

I*k*-ACM bases on the *k*-Anonymous Clustering Method given in [9]. In terms of complexity analysis, they do not differ with each other. In this subsection, complexity analysis is described as in [9].

Suppose that I*k*-ACM works on an input consisting of *n* event records and each record has *m* attributes. All of the *m* has distinct *V* different attribute value. Initialization phase mainly calculates the initial distance matrix and the running time of this part is *O*(*n*^2^.*m.V*). Initially there are *n* clusters and at the end of *k_n_*-anonymization phase, the minimum number of clusters is *n/k_n_*. Therefore, *n* − *n/k_n_* cluster combination operation occurs. Cluster combination consists of finding the minimum distance in the distance matrix and matrix reorganizing so that the distance values of new cluster are added and distance values of previous clusters are removed. If binary heap structure is used for finding minimum distance, formation of initial min heap structure with *n*^2^ elements is *O*(*n*^2^). In a heap, finding the minimum operation is *O*(1). However, removing distances of merged clusters from heap and adding the distances of new cluster to the heap need 2*n* deletion and *n* addition operations which cost *O*(*n* log(*n*)). Reorganization of distance matrix can be done in *O*(*n.m.V*) time sequentially with maintaining the heap. As a result, cost of each cluster combination operation is *O*(*nlogn* + *nmV*). Recall that maximum number of cluster combination operations is *n* − *n/k_n_*, the algorithm reaches to the end of *k_n_*-anonymization phase in *O*(*n*^2^*logn* + *n*^2^*mV*). Forming the output of I*k*-ACM takes *O*(*n* / *k_n_.m*). Formation of output does not change the overall running time. Totally, I*k*-ACM takes *O*(*n*^2^*logn* + *n*^2^. 2*mV*). *m* and *V* generally have lower values so they can be assumed as a constant factor. The running time can be fine-tuned to *O*(*n*^2^*logn*).

### 3.6. Multicasting and Energy Saving

Main aim of *k*-Anonymity solutions is providing the required privacy level with minimum information loss. However another factor, minimization of energy consumption, is an important criteria in WSNs. A sensor node consumes energy for different processes like event sensing, CPU processing, or transmitting/receiving data packets. Among these processes, transmission/reception operations consumes much of the energy. Table 3 shows energy consumption rates for transmission/reception which are published in technical report written by Carman *et al.* [12]. It is stated that energy consumption ratio of transmission/reception of one byte data to energy consumption of encryption of one byte data is 2333.34. Since each sensor node acts as a router for the messages of other nodes and one message goes over many hops in the network, energy saving for transmission/reception operations becomes a crucial design criterion. Shortening the length of messages and decreasing the number of travelled hops would help to reduce energy enormously.

**Figure 7.**
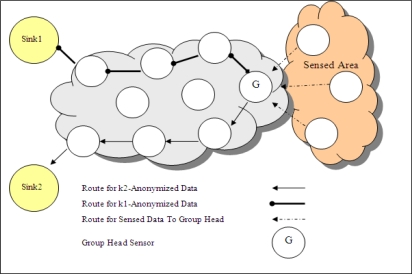
Routes when multiple *k*-anonymized outputs are generated.

**Figure 8.**
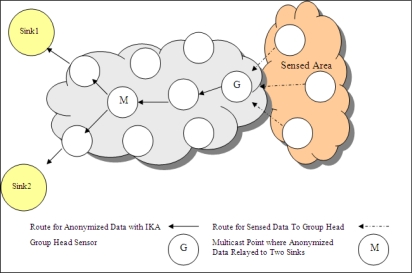
Routes when I*k*-ACM anonymized output is multicasted to sinks.

In a WSN topology where there are multiple sinks and each sink has different privacy criteria, the basic solution of anonymization is that group head sensor anonymizes the data, produces different outputs for each requirement of sink and sends each output to related sink in different paths as shown in Figure 7 (in this figure, there are two sinks in WSN). This sending method is called as *multipath*. However, I*k*-ACM produces unique output which is ready for multicasting. One anonymized output that guarantees all the privacy requirements, is sent to a multicast point. After reaching to multicast point, one copy of data is sent to sink1 and the other copy is sent to sink2 as presented in Figure 8. Multicasting schema decreases the number of travelled hops for some group head nodes.

Assume that there are two sinks named *Sink*1, *Sink*2. *Sink*1 requires *k*1-anonymized data and *Sink*2 requires *k*2-anonymized data. The lengths of anonymized outputs are *l_k_*_1_ and *l_k_*_2_, which are obtained by *k*-anonymization with only generalization operation for having *k*1-anonymized and *k*2-anonymized data, respectively. Data length of anonymous data generated by I*k*-ACM is labelled as *l_IkACM_*. The number of hops in the shortest route from group head sensor, *G*, to Sink1 and Sink2 is represented as *h_G,Sink_*_1_, *h_G,Sink_*_2_ respectively. Also assume that the hop distance between *G* and multicast point, *M*, is *h_G,M_*, distances from *M* to Sink1 and Sink2 are *h_M,Sink_*_1_ and *h_M,Sink_*_2_, respectively. Unique anonymized data is sent to, *M*, if an appropriate node exists in the network which holds the Inequality 5. Among the possible node candidates, the one which minimizes the value of *h_G,M_* + *h_M,Sink_*_1_ + *h_M,Sink_*_2_ is chosen.

(5) $$\left(h_{G,\mathrm{Sink}1}.l_{k1}\right)+\left(h_{G,\mathrm{Sink}2}.l_{k2}\right)>\left(h_{G,M}+h_{M,\mathrm{Sink}1}+h_{M,\mathrm{Sink}2}\right).l_{\mathrm{IkACM}}$$

If there is no appropriate *M* point, group head nodes prepare output for each sink and send them in different paths to sinks by multipathing. Location of sinks and location of group head sensor effect the selection among multicasting and multipathing.

Inequality 5 determines the level of energy consumption of multicasting and multipathing methods since it includes number of hops and the length of messages. It is assumed that the location of sinks are known by group head nodes and they are able to calculate the above inequalities with the routing information they have. Also they form the outputs for multicasting and multipathing, calculate the lengths of outputs, determine and use the best one in terms of energy saving.

## 4. Performance Evaluation of I*k*-ACM

In this part, information loss and energy saving trade-off is deeply investigated. Since the effectiveness of multicasting depends on the locations of sinks and distribution of sensor nodes, different experiments with different WSN topologies are performed.

Simulations are done by program that we developed in java. This program generates synthetic data with five attributes which are all categorical. Each categoric attribute has five distinct attribute values. I*k*-ACM is implemented and synthetic data is *k*-anonymized. In experiments, WSNs with different topologies are simulated and energy ratio calculations are performed according to Equation 7. Running of experiments are performed in a laptop having 1.20 Ghz CPU and 2GB RAM.

First experiment investigates the effect of sink locations. The size of WSN field is set to 500m × 500m. Distance of each hop, *R*, is taken as 10 m. There are two sinks in the field, *sink*1 and *sink*2. It is assumed that each sensor is uniformly distributed through the region. In every region having size of 10m × 10m, there is one group head sensor node. All the sensors convey their readings to their group head nodes. Then, they anonymize data and relays it to the sinks. Each group head node calculates the cost of multipathing and multicasting for a given set of data, chooses the best method for data sending.

The number of hops from sensor node to group head node and from group head node to sinks are calculated and they are taken into consideration in calculation of energy consumption. Energy consumption does not only depend on the number of hops; length of the messages are also important for the final results. Message lengths are taken into account during energy calculations.

Assume that energy consumption of method named as “multipath method” is denoted as *E_multipath_*. Energy consumption of method that uses multicasting when appropriate is represented as *E_hybrid_*. Energy saving ratio, *ES*, is computed as follows:

(6) $$\mathrm{ES}=1-\frac{E_{\mathrm{hybrid}}}{E_{\mathrm{multipath}}}$$

(7) $$\mathrm{ES}=1-\frac{\sum_{\mathrm{for}\cdot \mathrm{all}\cdot G}\text{min}((h_{G,\mathrm{Sink}1}.l_{k1})+(h_{G,\mathrm{Sink}2}.l_{k2}),((h_{G,M}+h_{M,\mathrm{Sink}1}+h_{M,\mathrm{Sink}2}).l_{\mathrm{IkACM}}))}{\sum_{\mathrm{for}\cdot \mathrm{all}\cdot }\left(h_{G,\mathrm{Sink}1}.l_{k1})+(h_{G,\mathrm{Sink}2}.l_{k2}\right)}$$

Values of *k*1, *k*2 are chosen as 3 and 6 respectively. Input records have five attributes each having distinct four attribute values. The range of information loss is computed as [0.0 2.0] in this experiment. If all group head nodes use multipath method, information loss for the data sent to *sink*1 is found as 0.44 and data loss for *sink*2 is 0.88. Total information loss of the whole system is 0.66 for multipath method. If multicasting with I*k*-ACM is used when appropriate, information loss for *sink*1 also does not change since the first step of I*k*-ACM is a normal *k*1-anonymization step. However, information loss of *sink*2 and energy saving ratios change. Table 4 gives the results obtained for different sink locations. Five different locations as given in the first column are chosen. Second column presents total information loss occurred in the system. The number of nodes using multicasting and multipathing methods are shown in third and fourth columns. Last column gives the result of energy saving according to the case when all nodes are using multipathing method.

As the sinks get closer to each other, it is observed that the number of group head nodes using multicasting increase due to finding optimum multicast points. In the case where the sinks are located in coordinates of (0,0) and (500,500), sinks have the maximum distance between each other. 716 group head nodes, out of 2500 nodes, choose multicasting as the best alternative. Information loss increases to 0.73 bits but energy gain is very limited, 3%. In this topology, the number of multicasting nodes is lower and energy consumptions of hybrid and multipathing methods do not differ. If sinks are located in coordinates (200,0) and (300,0), 2406 nodes choose multicasting. In this case, energy saving increases to 32% and information loss increases to 0.90. Actually, there is a trade-off between data utility and energy consumption. If there is a need for energy minimization and some sinks can tolerate additional data losses, using multicasting when appropriate in some network topologies may be an efficient solution.

**Figure 9.**
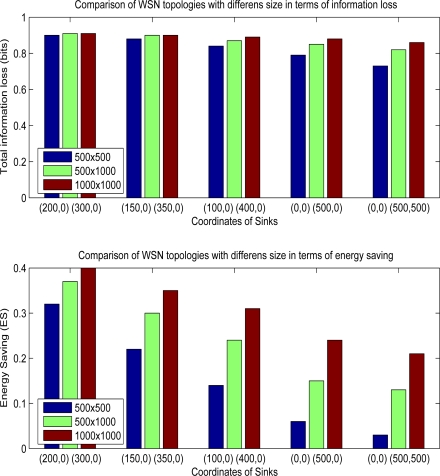
Performance comparison of topologies with different WSN area sizes.

Other than the location of sinks, another parameter that may effect the efficiency of hybrid method is the total size of WSN field. Figure 9 shows the overall performance results of WSN fields having different sizes and different sink locations. As the size of sensor field increases, hybrid method makes the network consume less energy. For example, when the sink coordinates are (0,0) and (0,500), energy saving is 0.22 for WSN size, 500 m × 500 m, however gain rises to 0.35 for mode wider size, 1,000 m × 1,000 m. Since, the distance between each sink is not different in both situations, ratio of group head nodes which choose multicasting increases in wider WSN fields. In field size 500 m × 500 m, 1287 of 2500 group head nodes choose multicasting. On the other side, in field having sizes 1000 m × 1000 m, 8787 of 10000 group head nodes select multicasting option. The cost of increasing the energy saving is the lower data utility since information loss increases to 0.88 from 0.79 in 1000 m × 1000 m sized field. In experiments having sink locations like (0,0), (0,500) and (0,0), (500,500), energy savings are nearly 3–6 sized fields which seem that hybrid method does not create any promising results. However, in wider sized area like 1000 m × 1,000 m, hybrid method yields good results so that energy saving is above than 20%, If the sink locations are (200,0) and (300,0) for a WSN size 1,000 m × 1,000 m, energy saving is 40% which is maximum gain among the all experiments. Total information loss rises to 0.91 from 0.66 if we compare hybrid with multipathing.

## 5. Related Work

Studies on privacy problem mostly concentrated on achieving sharing of databases under the required privacy constraints in order to make efficient knowledge-based decisions. Generic name, “Privacy Preserving Data Publishing”, is given to these efforts [4]. *k*-anonymity notion is introduced by Samarati and Sweeney in [5]. It is shown that *k*-anonymization with minimum number of suppression is NP-hard [13]. Some optimal *k*-anonymization algorithms have been presented which may be feasible for small sized data sets [14,15]. Greedy heuristics algorithms are proposed to find approximate solutions for large data sets [16,17].

All these *k*-anonymity solutions solve the prevention of “record linkage attack” which is actually finding the owner of a record through quasi-identifier attributes. However, it is shown that without finding the exact owner of a record, if sensitive attribute exists in a record, it may be possible to identify sensitive attribute of an individual in some circumstances by an attack called “attribute linkage attack” [4]. This problem is also named as “attribute disclosure” [18]. In order to prevent attribute linkage and record linkage together, *k*-anonymity notion extended in some studies. Machanavajjhala *et al.* extended *k*-anonymity with a *l*-diversity notion that also prevents the identity disclosure when attackers have background knowledge [18]. Notion of *p*-sensitive is introduced so that *p* of *k*-anonymized records having identical quasi-identifier attribute values have to have distinct sensitive attribute values [19]. Generalization hierarchies are constructed for sensitive attributes and extended version of *p*-sensitive notion is adapted in [20]. An additional requirement, *t*-closeness, for *l*-diversity is defined in [21]. In this study, distribution of sensitive attributes in a record set having identical quasi-identifier attribute values are adjusted so that it is close to the distribution of that attribute in overall data set.

Anonymity is considered as hiding the identities of sender or receiver of a communication in data and communication networks for many years. DC-Net and mix-net solutions are proposed for achieving sender or receiver anonymity [22,23]. Especially, mix-net idea have been used in many practical Internet applications like web and e-mail [24,25]. In ad-hoc networks, routing protocols for anonymous transmission of the data packets are designed.

Studies about the anonymity problem in WSNs basically try to hide location or time information of the events. Gruteser *et al.* [26,27] proposed anonymity solutions for providing high degree of privacy in a sensor network that gives location-based services. Ozturk *et al.* [28] proposed phantom routing method for hiding location information of originator sensor node in a sensor network. Threat model is based on an existence of only one movable adversary node in the environment. Location privacy protection of receiver in a WSN is provided by a routing protocol in [29]. Proposed routing protocol prevents the eavesdropper to identify the receiver by tracing the wireless packets. It randomizes the routing paths and injects fake packets in order to mislead eavesdroppers. Wadaa *et al.* [30] studied on providing anonymity of coordinate system, cluster and routing structures during the network setup of a WSN. Protection of location privacy is guaranteed by *k*-anonymity in location based services those are given on mobile networks [31].

Privacy studies of WSN community generally focuse on hiding sender or receiver entities from local or global eavesdroppers. However, in data collection applications, sender or receiver entities are known by all parties. Privacy threat models of data collection applications should concentrate on privacy of collected data against data collectors rather than hiding the communicated entities. On the other side, the ones which deal with privacy of collected data propose solutions only for location and time data of events. An event may have other attributes which may violate the privacy of individuals.

In this paper, since the targeted applications are data collection ones, collected data is considered as the subject of privacy. All attributes those may help to distinguish an individual are considered during anonymization. Privacy is guaranteed against for the data collector parties which are actually sinks in WSN applications. Also, by the proposed method, it is possible to provide different privacy requirements of many sinks.

## 6. Conclusions

In this paper, privacy preserving data collection framework is proposed for WSNs. The network and threat model of framework states that there exists multiple sinks and each sink has different level of privacy requirements.

In order to meet the requirements of network and threat model, we propose a method called I*k*-ACM (Iterative *k*-Anonymization Clustering Method). Proposed I*k*-ACM reduces energy consumption while fulfilling the required different privacy levels of sinks.

I*k*-ACM Method uses encryption operations with generalization operations in order to have one common anonymized output. This common output enables us to multicast the same message to all sinks. Multicasting of this output enables WSN to reduce amount of energy consumed for transmitting it to the sinks. In WSN, each local region has one group head node. They gather event data from sensors of their local region, anonymize it and send it to the all sinks. According to the positions of group head nodes and their distances to sinks, multicasting can be better alternative for some of the group head nodes. Each group head node decides whether multicasting is appropriate for itself or not. If it decreases energy consumption, group head node uses it. Multicasting method degrades the quality of data gathered by some sinks. Here, there is a trade-off between data loss and energy consumption. WSN designers has to decide about the trade-off between energy saving and information loss. We analyze this trade-off for different sized WSN topologies and for different sink locations. Our analyses show that, in a WSN having two sinks, it is possible to save 32% of energy, however the loss of data utility received by one of the sink increases to 0.90 from 0.66 in some topologies.

## Figures and Tables

**Table 1.** A sample bit string representation set.

Records	*B*_*i*1_	*B*_*i*2_	*B*_*i*3_

*T*_1_	00010	01000	10000
*T*_2_	01100	11100	01111

**Table 2.** A sample normalized version of bit string representation set.

Records	$\bar{B_{i1}}$	$\bar{B_{i2}}$	$\bar{B_{i3}}$	

*T*_1_	00010	01000	10000
*T*_2_	0 $\frac{1}{2}$$\frac{1}{2}$00	$\frac{1}{3}$$\frac{1}{3}$$\frac{1}{3}$00	0 $\frac{1}{4}$$\frac{1}{4}$$\frac{1}{4}$$\frac{1}{4}$

**Table 3.** Energy consumption ratios.

**Energy Consumption Ratios**	**Ratio Value**

Transmission/Reception	1.5
Transmission/Encryption	2333.34
Encryption/Decryption	1

**Table 4.** Results of using hybrid and multipathing together with different sink locations.

**Location of Sinks (coordinates)**	**Total Info Loss For (bits)**	**Number of Group Head Nodes Using Multicasting Method**	**Number of Group Head Nodes Using Multipathing Method**	**ES** (%)

(0,0),(500,500)	0.73	716	1784	3
(0,0),(500,0)	0.79	1287	1213	6
(100,0),(400,0)	0.84	1813	687	14
(150,0),(350,0)	0.87	2137	363	22
(200,0),(300,0)	0.90	2406	94	32
